# Supplementary material for: “It’s just an issue and you deal with it… you just deal with it, you move on and you do it together.”: Men’s experiences of bacterial vaginosis and the acceptability of male partner treatment
Source: PLoS One. 2020 Jun 29;15(6):e0235286. doi: 10.1371/journal.pone.0235286 (PMC7323956; doi:10.1371/journal.pone.0235286)
Supplement: S1 Interview schedule — (PDF) [file pone.0235286.s001.pdf]

S1 Interview schedule.

These questions are intended as a guide only and may be amended as lines of questioning emerge.

<u>Demographic questionnaire</u>

1. **Age:**

2. **Postcode:**

3. **Country of birth:**

4. **Ethnic background** *(i.e. your parents birthplace/s)*……………………………………………………………

5. **Highest level of formal education <u>you have completed</u>:**
   Up to year 10 ◯ Year 12 ◯ Diploma/TAFE ◯ Undergraduate degree ◯ Post graduate degree ◯ Other ◯

6. **Gender assigned at birth :**
   Male ◯ Female ◯

7. **Have sex with:**
   Men ◯ Women ◯ Both ◯ Other: Please specify ……………

8. **I identify as**:

   Heterosexual ◯ Gay ◯ Bisexual ◯ Queer ◯ Other ◯

9. **Prior to participating in Step Up RCT, had you ever been tested for an STI?**
   Yes ◯ No◯

10. **What method do you and your partner use as contraception**

    The pill ◯ IUD ◯ Implanon ◯ Condoms ◯

    Nuva ring ◯ Withdrawl ◯ Other (please specify) ◯ ……

11. **Any BV recurrence since study participation?**
12. **How long have you been with your partner?**
13. **Did you receive treatment?**

<u>Interview</u>

So, tell me about how you found out about the trial?

Knowledge and previous experience of BV

- What did you know about BV before the trial?
- What do you know about BV after the trial?
- Were you aware your partner had BV? How often was she getting BV?
    - Any signs or symptoms you noticed that you thought may have been unusual?
    - Any previous sexual partners w BV or symptoms?
- How has BV affected your partner?
- How has BV affected you?
- How has BV affected your relationship?
    - Sex life/ Anything you and your partner do differently because of BV?
- Have you noticed any changes in your partner since treatment?
- Have you noticed any changes in yourself post treatment?

BV as an STI (informed by some questions in demographic portion)

- Had you ever been tested for an STI prior to the trial?
- What prompted this?
- Do you think STIs are a "big deal"? (i.e. Serious health consequences)
- How would you feel if you were diagnosed with an STI?
- What are your thoughts on BV being an STI?
- Do you think BV is a "big deal"? (ie. Serious infection/issue for the relationship)
  How do you feel about the diagnosis of BV? Would you feel any different if it were classified as an STI?

Reasons for participation

- Why did you participate in the study?

  Any concerns/hesitations? How resolved?

Experience of study and treatment

- How did you find the study? (amount of contact/how engaged?/educational)
- How did you find the treatment? Oral vs topical. (SEs, affect on lifestyle, adherence- if only one, which prefer?) Anything could improve this? (timing of treatment, etc)

Hypothetical

- What do you think might make some guys accept/decline participating in the study?

- If partner treatment were routine, rather than part of a study, do you think this might make men more inclined to take treatment? Why/not?

    - Is there anything that might influence you to accept/decline treatment- eg. casual partner, your experience of treatment

- There's currently no diagnostic for men to confirm BV. How do you think this might affect your willingness to accept partner treatment?
- How has participating in the study and taking treatment affected your relationship? (positively/negatively) (i.e. Opened up communication around sexual health/ partner grateful)

Receiving information

- Some people can find having the conversation about STIs and partner treatment uncomfortable. Is there anything that would help you have this conversation with your partner?

-If male partner treatment was show to be effective and you had a partner with BV, would you prefer to receive treatment and information directly from your partner, or a clinician?
